# Cluster analysis of plasma cytokines identifies two unique endotypes of children with asthma in the pediatric intensive care unit

**DOI:** 10.1038/s41598-023-30679-9

**Published:** 2023-03-02

**Authors:** Kirsten A. Cottrill, Milad G. Rad, Michael J. Ripple, Susan T. Stephenson, Ahmad F. Mohammad, Mallory Tidwell, Rishikesan Kamaleswaran, Anne M. Fitzpatrick, Jocelyn R. Grunwell

**Affiliations:** 1 Department of Pediatrics, Emory University School of Medicine, Atlanta, GA USA; 2 Division of Critical Care Medicine, Children’s Healthcare of Atlanta at Egleston, 1405 Clifton Road NE, Atlanta, GA 30322 USA; 3 Department of Electrical and Computer Engineering, Georgia Institute of Technology, Atlanta, GA USA

**Keywords:** Cytokines, Gene regulation in immune cells, Asthma

## Abstract

Children with life-threatening asthma exacerbations who are admitted to a pediatric intensive care unit (PICU) are a heterogeneous group with poorly studied inflammatory features. We hypothesized that distinct clusters of children with asthma in a PICU would be identified based on differences in plasma cytokine levels and that these clusters would have differing underlying inflammation and asthma outcomes within 1 year. Plasma cytokines and differential gene expression were measured in neutrophils isolated from children admitted to a PICU for asthma. Participants were clustered by differential plasma cytokine abundance. Gene expression differences were compared by cluster and pathway over-representation analysis was performed. We identified two clusters in 69 children with no clinical differences. Cluster 1 (*n* = 41) had higher cytokines compared to Cluster 2 (*n* = 28). Cluster 2 had a hazard ratio of 2.71 (95% CI 1.11–6.64) compared to Cluster 1 for time to subsequent exacerbation. Gene expression pathways that differed by cluster included interleukin-10 signaling; nucleotide-binding domain, leucine rich repeat containing receptor (NLR signaling); and toll-like receptor (TLR) signaling. These observations suggest that a subset of children may have a unique pattern of inflammation during PICU hospitalization that might require alternative treatment approaches.

## Introduction

Asthma is the most common chronic lung disease of childhood, affecting approximately 5–8% of persons under the age of 18 years in the United States (U.S.)^1,2^. Yet despite advances in asthma care, asthma remains poorly controlled in the majority of affected children. The most recent healthcare use data from the Centers for Disease Control and Prevention National Health Interview Survey estimate that 104.7 per 10,000 U.S. children under the age of 18 years are seen in an Emergency Department for an asthma attack each year, with approximately 10% of these children hospitalized^3–5^. Furthermore, asthma accounts for 3.5–5% of annual Pediatric Intensive Care Unit (PICU) admissions in the U.S.^6^. Children admitted to the PICU for life-threatening asthma are a vulnerable group and frequently have a shorter time to hospital readmission for asthma compared to children without prior PICU admissions^7^, which may reflect underlying disease that is refractory to conventional asthma treatment.

It is increasingly recognized that asthma is a heterogeneous disorder with high morbidity^8–10^. Although advances have been made in defining clinical “phenotypes” (i.e., groups shared biological inflammatory mechanisms) in pediatric outpatient populations, this work has not extended to children with life threatening asthma who are admitted to the PICU. This is a major shortcoming since children admitted to the PICU for asthma vary in their age, response to treatment, chronic asthma severity classification, sensitivity to triggers, and exacerbation frequency^11,12^. For example, in one study, up to 16% of children who presented to the PICU with life threatening asthma were experiencing their first major asthma exacerbation and would be considered as having “mild” asthma in the outpatient setting^13,14^.

Given the profound lack of mechanistic insight in children with life-threatening asthma admitted to the PICU, we examined cytokine networks and gene expression pathways in children ages 6–17 years admitted to a PICU for asthma. We hypothesized that distinct clusters of children with life-threatening asthma would be identified based on differences in plasma cytokine levels and that these clusters would have differing clinical features at presentation to the PICU and differing asthma outcomes within 1 year of PICU admission. We then performed an exploratory analysis of the gene expression networks of differentially expressed neutrophil genes within the plasma-cytokine-defined clusters. We hypothesized that novel differentially expressed gene networks would be associated with cluster assignment.

## Methods

### Patient cohort

We performed a single-center, prospective, observational cohort study of children between the ages of 6–17 years who were admitted to the Emory University-affiliated Children’s Healthcare of Atlanta 36-bed PICU for severe acute asthma between July 29, 2019, and February 10, 2021. The Emory University School of Medicine Institutional Review Board (IRB00110747) approved the study. Informed consent was obtained from all participants and/or their parent or legal guardian prior to enrollment. In accord with institutional requirements, we obtained verbal assent from children 6–10 years and written assent from children 11 years of age or greater prior to enrollment and any study procedures. All study procedures were performed according to the relevant guidelines and regulations in the Declaration of Helsinki. Children are admitted to the PICU for asthma if they received any of the following interventions in the Emergency Department: (1) a third continuous nebulized albuterol treatment, (2) non-invasive respiratory support delivered by high-flow nasal cannula or bilevel positive airway pressure, (3) intubation for invasive mechanical ventilation, (4) receipt of a 80%/20% helium–oxygen mixture for hypoxemia, or (5) required greater than or equal to 50% fraction of inspired oxygen by Venturi mask or positive pressure ventilation to maintain oxygen saturations ≥ 92%. Children were excluded if they had other chronic medical conditions requiring systemic corticosteroids or had disorders necessitating immunosuppressive medications such as a history of hematopoietic stem cell or solid organ transplant, oncologic diagnoses, sickle cell anemia, or rheumatologic diagnoses. Children with respiratory comorbidities such as cystic fibrosis, pulmonary aspiration, gastroesophageal reflux requiring acid suppression medication and/or tube-feed dependence, bronchiectasis, congenital airway anomalies, bronchopulmonary dysplasia, and/or a history of premature birth before 35-week gestation were excluded. Pregnant patients and those with a personal history of any recreational smoking or vaping were also excluded.

### Characterization procedures and longitudinal outcomes

Characterization procedures were performed during the PICU admission. Medical history and treatments received were obtained from the Electronic Medical Record (EMR). Asthma control in the 4 weeks prior to hospitalization was measured using the validated Childhood-Asthma Control Test (C-ACT) for those children between 6–11 years of age and the ACT for those children 12–17 years of age. A C-ACT or ACT score of 19 or less indicates uncontrolled asthma^15–17^. Longitudinal asthma outcomes were extracted from the EMR.

### Blood collection and sample processing

Blood was collected from participants during their PICU admission through an existing peripheral intravenous catheter with blood return or a central venous catheter into an ethylenediaminetetraacetic acid (EDTA) vacutainer tube. Tubes were centrifuged at 400ΧG to separate cells from platelet-rich plasma. Pelleted blood cells were resuspended in sterile phosphate buffered saline (PBS; ThermoFisher Scientific, Waltham, MA) with 2.5 mM EDTA up to the original whole blood volume. Neutrophils were negatively selected from PBS-EDTA washed whole blood using the EasySep Direct Human Neutrophil Isolation kit (StemCell Technologies, Cambridge, MA) according to the manufacturer’s protocol. Neutrophil purity was > 99% as confirmed by microscopic analysis of a Diff-Quik stained CytoSpin sample of purified neutrophils. Two million neutrophils were resuspended in 1 mL of RNALater and stored at − 80 °C until batch RNA purification.

### Cytokine measurement

Plasma was stored at − 80 °C and analyzed in a single batch on a Luminex MAGPIX system (Millipore) according to the manufacturer’s protocol. A 21-plex human T cell Panel array (HSTCMAG 28SK, T cell Panel, Millipore) was used to quantify fraktaline; granulocyte–macrophage colony-stimulating factor (GMCSF); interferon gamma (IFNG); interleukins (IL) 1b, 2, 4, 5, 6, 7, 8, 10, 12 (p70), 13, 17, 21, 23; interferon–inducible T cell alpha chemoattractant (ITAC); macrophage inflammatory protein (MIP) 1A, 1B, 3A; and tumor necrosis factor alpha (TNFa) as previously published by our group comparing obese to non-obese children with asthma who were not having an exacerbation^18^.

### RNA preparation

RNA was isolated from neutrophils using the Nucleospin RNA II kit with on-column genomic DNA digestion according to the manufacturer’s protocol (Takara, Mountain View, CA). The Emory Integrated Genomics Core performed RNA sizing quantification and quality control using Pico and Nano Agilent kits with an Agilent 2100 bioanalyzer. The concentration of RNA was measured using a Tecan optical density plate reader^12^.

### NanoString array

We measured differential gene expression of 579 genes of interest from purified neutrophils using the Human Immunology v2 NanoString nCounter Gene Expression CodeSet (NanoString, Seattle, WA). All NanoString-based measurements were conducted at the Emory University Integrated Genomics Core facility using an amplification step for low abundant RNA applied to all samples as previously described^18^. The raw data are available at Gene Expression Omnibus (https://www.ncbi.nlm.nih.gov/geo/query/acc.cgi?acc=GSE208220).

### Cluster assignment procedure

**Figure 1:**
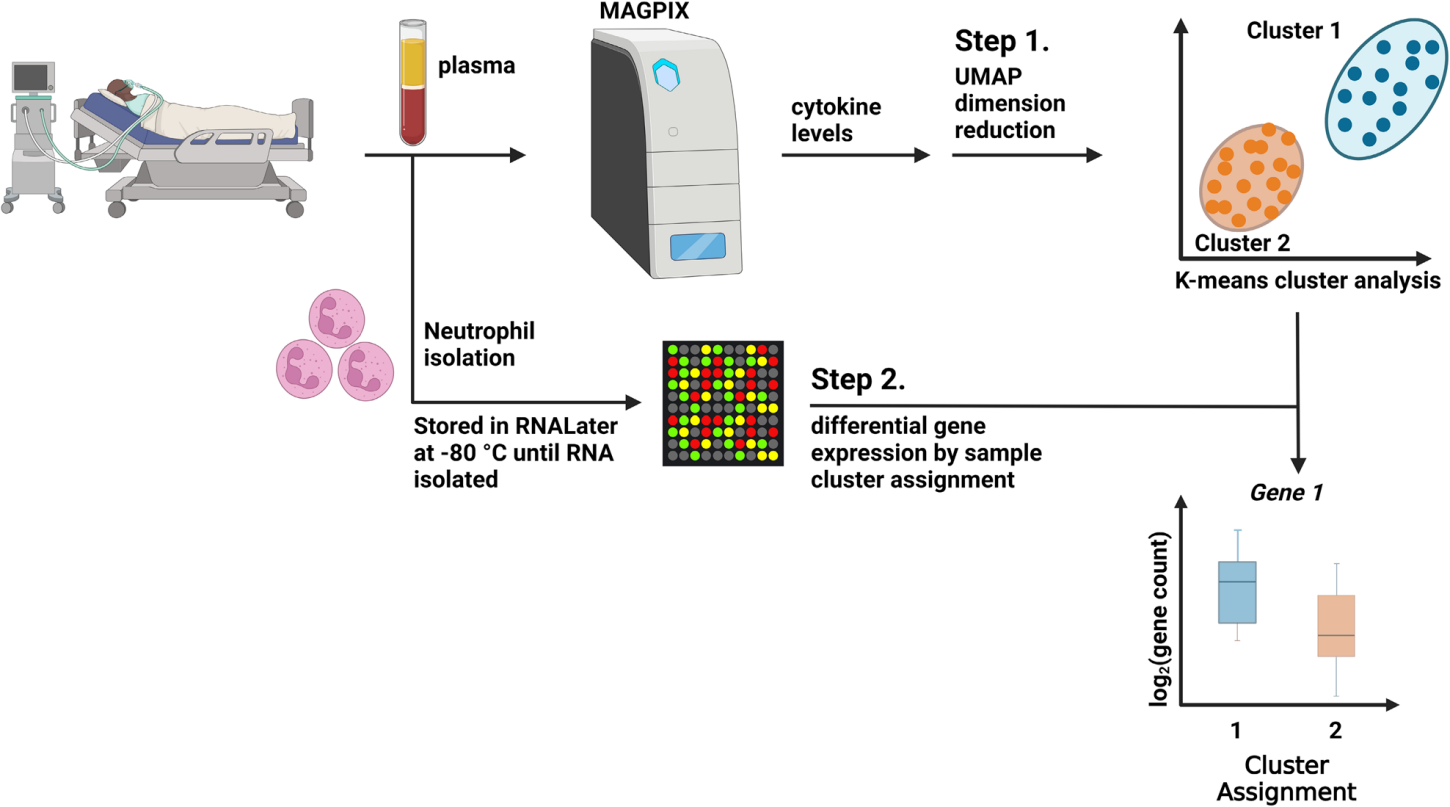
Experimental overview and sample processing. Plasma cytokines were analyzed on a Luminex MAGPIX system (Millipore) using a 21-plex human T cell Panel array (HSTCMAG 28SK, Millipore) according to the manufacturer’s protocol. Following UMAP dimension reduction (Step 1), k-means clustering was used minimize within-cluster sum of squares and Silhouette analysis was performed to yield two clusters of differentially expressed plasma cytokines. Neutrophils were also isolated by negative magnetic bead selection from children ages 6–17 years admitted to the pediatric intensive care unit for an asthma exacerbation, resuspended in RNALater, and stored at − 80 °C until analysis using the Human Immunology v2 NanoString nCounter Gene Expression CodeSet. In Step 2, the differential gene expression of baseline neutrophils was analyzed using the two clusters defined by the plasma cytokine cluster analysis shown in Step 1. Created with BioRender.com with a confirmation of publication and licensing rights.

Clustering was performed on the plasma cytokine abundances using the Python scikit-learn package^19^. We used the uniform manifold approximation and projection (UMAP) python library for visualization of the clusters^20^. We assigned cluster membership using the spectral clustering algorithm within the scikit-learn library^19^. The spectral clustering algorithm generates clusters using graph theory and connected edges. The number of clusters were determined by the cross-validation method. An overview of the sample preparation and experimental study design is shown in Fig. 1.

### Differential gene expression and pathway analysis by cluster assignment

We performed an over-representation gene network analysis of the differentially expressed genes using Reactome^21,22^.

### Statistical analysis

Only children with complete cytokine data were included in the clustering analysis. Normality of features was evaluated by Shapiro–Wilk tests. Differences in demographic and clinical features were assessed using *t* tests for normally distributed continuous variables, Kruskal–Wallis for non-normally distributed continuous variables, and chi-square or Fisher’s exact test for categorical variables depending on the number of subjects per category. There were 59 children with BMI percentiles available for analysis. We defined obesity as having a BMI ≥ 95th %-ile. Time to exacerbation was modeled using a Cox proportional hazard model with cluster identity as the independent exposure variable with and without adjusting for obesity as a confounding factor using the R package survival^23^. The proportional hazards assumption was checked by visual inspection of log–log plots and analyzing the Schoenfeld residuals. Differences in cytokines were assessed using Mann–Whitney *U* tests to account for non-normal distributions. Differences in gene expression were assessed using *t* tests. The Benjamini–Hochberg method was used to correct for multiple comparisons^24^. A *p*-value of less than 0.05 and a *q*-value of less than 0.1 was considered significant.

## Results

### Study population

We enrolled 96 children into the study at the time of analysis. We were not able to obtain blood from 18 children due to no blood return from a peripheral intravenous line and a refusal of a separate lab draw. An additional 9 children did not have enough plasma volume to complete cytokine analysis, yielding a final sample of 69 children with complete data for analysis (Supplementary Fig. 1). The demographic and asthma history of the 69 children in the final cohort are summarized in Table 1.

**Table 1:** Demographics and asthma history of the participants by cluster.

Characteristic, n (%)	Cluster 1 *n* = 41	Cluster 2 *n* = 28	*p-*value
Age, yr, median (Q1, Q3)	11.0 [9.0,14.0]	9.5 [7.8,14.0]	0.495
Sex: female/male	15 (37)/26 (64)	13 (46)/15 (54)	0.570
BMI percentile, mean (SD)^a^	59 (35)	84 (23)	0.002
Self-identified race			
Black	34 (83)	25 (89)	0.861
White	3 (7.3)	1 (3.3)	
American Indian	1 (2.4)	0 (0)	
Multi-racial or other	1 (2.4)	1 (3.6)	
Self-identified ethnicity	1 (1.4)	1 (3.6)	0.393
Insurance			
Private	7 (17.5)	7 (25)	0.601
Self-pay	1 (2.5)	0 (0)	
Government/Medicaid	31 (77.5)	21 (75)	
Military/Tricare	1 (2.5)	0 (0)	
History of asthma prior to PICU^b,c^	35 (87.5)	25 (89)	> 0.9
Asthma Control Test, mean (SD)^d^	16 (6.2)	16 (4.2)	0.520
Home asthma medications^e^			
None	5 (12)	4 (14)	> 0.9
Albuterol or Xopenex	26 (63)	18 (64)	> 0.9
Montelukast	12 (29)	14 (50)	0.136
ICS	13 (32)	10 (36)	> 0.9
ICS + LABA	10 (24)	9 (32)	0.665
Omalizumab	1 (2.4)	2 (7.1)	0.562
Daily oral corticosteroids	2 (2.9)	2 (7.1)	0.161
Daily use of β-agonist (at least 5/7 days)^f^	12 (34)	13 (52)	0.268
In past year, asthma resulted in:^e,f^			
Emergency/urgent care visits	17 (49)	19 (76)	0.061
Hospitalized in PICU	4 (11)	7(28)	0.176
In lifetime, asthma resulted in:^e,f^			
Hospitalized in PICU	14 (40)	12 (48)	0.725
Intubated for asthma	4 (11)	2 (8.0)	> 0.9
Medical history^e^			
Allergic rhinitis	9 (22)	8 (29)	0.732
Eczema	22 (54)	18 (64)	0.529
Sinusitis	1 (2.4)	0 (0)	> 0.9
Family asthma history^e^			
No one else has asthma	11 (27)	5 (18)	0.564
Father	10 (24)	10 (36)	0.455
Mother	13 (32)	10 (36)	0.931
Sibling	10 (24)	6 (21)	> 0.9
Indoor exposures^e^			
None	14 (34)	5 (18)	0.225
Tobacco smoke	15 (37)	8 (29)	0.665
Pets	14 (34)	10 (36)	> 0.9

^a^Ten missing in Cluster 1, 2 in Cluster 2, ^b^Pediatric intensive care unit; ^c^One Missing in Cluster 1; ^d^7 missing in Cluster 1, 6 in Cluster 2; ^e^More than one condition can be selected, and frequency total may add up to more than 100%; ^f^Six missing in Cluster 1, 3 in Cluster 2.

### Cluster assignment by plasma cytokines

**Figure 2:**
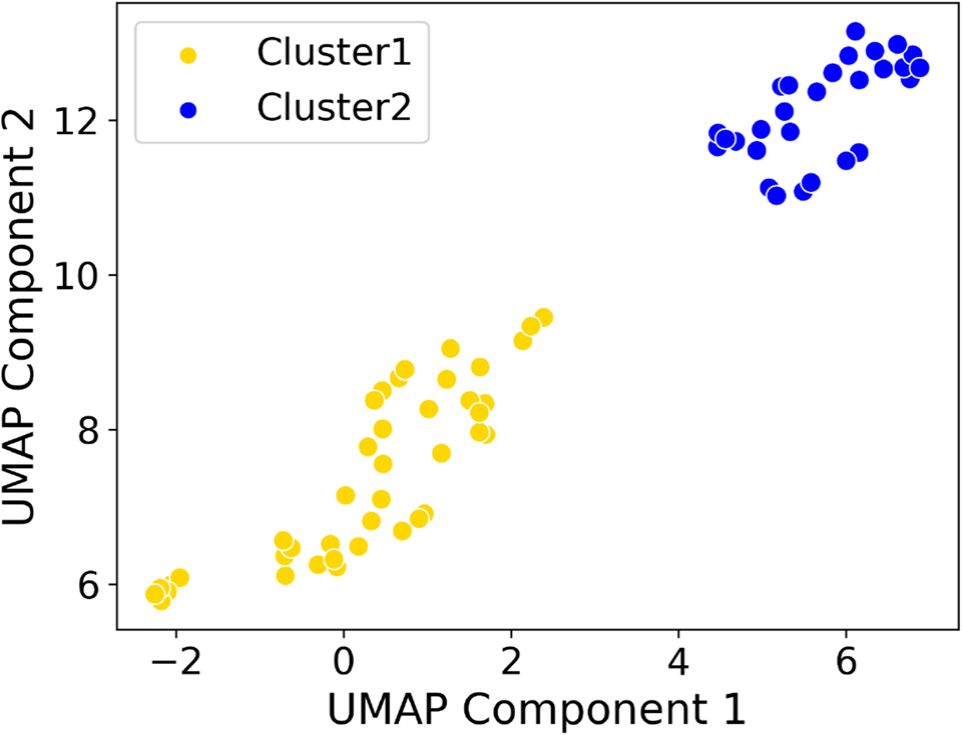
Uniform Manifold Approximation and Projection (UMAP) of component 1 versus component 2 showing two clusters (Cluster 1 = gold, *n* = 41; Cluster 2 = blue, *n* = 28) of participants with differentially expressed cytokines identified by spectral clustering.

We first used the 21 available cytokine concentrations to cluster these children into two groups as shown in Fig. 2. There were 41 children assigned to Cluster 1 and 28 children assigned to Cluster 2. Individual cytokines that distinguished the Clusters are shown in Table 2. Overall, there were significantly higher cytokine concentrations in Cluster 1 compared to Cluster 2, with the exception of interleukin-8 (IL-8). MIP-1A had a lower median concentration in Cluster 1 compared to Cluster 2; however, due to a right-sided skew, the mean MIP-1A concentration was higher in Cluster 1 compared to Cluster 2. There were essentially no differences in demographic characteristics or asthma history between clusters, although Cluster 2 had a significantly higher BMI percentile (mean [SD], 84% [23%]) compared to Cluster 1 (mean [SD], 59% [35%]) (Table 1). There were no differences in treatment received prior to admission, treatment in the Emergency Department or PICU, or blood cell counts by cluster (Supplementary Table 1).

**Table 2:** Plasma cytokine concentrations.

Cytokine median (Q1, Q3) mean [SD]	Cluster 1 (pg/mL) *n* = 41	Cluster 2 (pg/mL) *n* = 28	*p*-value
ITAC	19.29 (13.41, 27.09) 22.01 [13.00]	8.67 (6.45, 13.88) 19.65 [48.47]	< 0.0001
GM-CSF	49.15 (37.64, 85.84) 75.54 [67.39]	24.2 1 (12.79, 41.15) 36.07[42.72]	< 0.0001
Fractalkine	131.83 (112.95, 151.36) 136.04 [35.94]	90 (72.86, 117.74) 96.65 [35.09]	< 0.0001
IFN-γ	14.79 (12.73, 18.39) 15.21 [4.78]	4.94 (3.25, 6.09) 4.94 [1.95]	< 0.0001
IL-10	26.54 (16.66, 35.38) 30.26 [21.35]	10.15 (8.26, 14.02) 14.79 [17.14]	< 0.0001
MIP-3A	20.79 (8.46, 93.66) 25.67 [16.50]	6.43 (3.1, 15.26) 7.38 [3.41]	< 0.0001
IL12-p70	2.19 (1.78, 2.67) 2.32 [0.90]	0.96 (0.65, 1.21) 0.96 [0.37]	< 0.0001
IL-13	3.3 (1.54, 12.49) 10.77 [17.13]	1.39 (0.98, 2.13) 3.14 [5.08]	0.0008
IL-17	11.02 (8.37, 13.28) 11.18 [4.16]	3.81 (2.92, 5.04) 4.10 [1.61]	< 0.0001
IL-1β	1.69 (1.25, 1.89) 1.69 [0.69]	0.365 (0.26, 0.60) 0.49 [0.32]	< 0.0001
IL-2	2.34 (1.82, 2.77) 2.44 [0.98]	0.8 (0.54, 1.25) 0.91 [0.47]	< 0.0001
IL-21	3.96 (2.71, 4.89) 4.47 [2.75]	1.485 (1.15, 2.16) 1.79 [1.02]	< 0.0001
IL-4	38.79 (27.54, 142.29) 99.55 [155.26]	8.03 (5.99, 14.33) 10.16 [6.20]	< 0.0001
IL-23	234.25 (161.49, 317.13) 270.25 [182.42]	128.59 (95.66, 173.82) 133.53 [60.19]	< 0.0001
IL-5	3.68 (2.8, 6.16) 4.99 [3.47]	2.39 (1.44, 3.65) 2.57 [1.42]	0.0001
IL-6	3.25 (1.86, 23.78) 17.55 [29.81]	1.26 (1.044, 2.79) 2.61 [3.38]	0.0003
IL-7	8.51 (6.74, 9.86) 8.41 [2.54]	3.13 (2.35, 3.89) 3.20 [1.04]	< 0.0001
IL-8	3.35 (2.12, 15.82) 14.71 [26.10]	3.07 (2.34, 3.95) 3.54 [2.08]	0.18
MIP-1A	7.07 (4.62, 11.36) 18.73 [44.49]	10.31 (8.37, 13.92) 10.85 [4.38]	0.02
MIP-1B	5.18 (3.56, 6.7) 5.63 [3.54]	2.33 (1.99, 3.21) 2.44 [0.93]	< 0.0001
TNF-α	3.43 (2.76, 4.24) 3.71 [1.57]	1.11 (0.98, 1.43) 1.23 [0.38]	< 0.0001

### Clinical outcomes by cluster assignment

**Figure 3:**
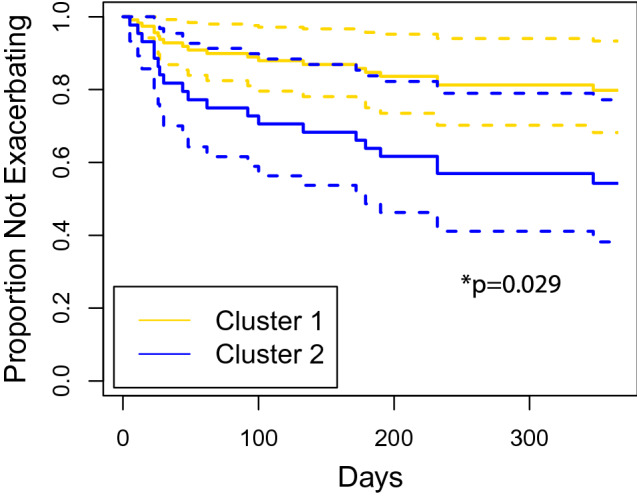
Cox proportional hazards model of time to subsequent exacerbation for Cluster 1 (gold, *n* = 41) and Cluster 2 (blue, *n* = 28) over the year following initial discharge. The 95% confidence intervals are represented by dashed lines. Cluster 2 had a hazard ratio of 2.71 (95% CI 1.11–6.64) compared to Cluster 1 (*p* = 0.029).

There was no difference in PICU or hospital length of stay, discharge location, or medications prescribed at discharge between the two clusters (Table 3). Longitudinal outcomes were available for 33 children in Cluster 1 and 23 children in Cluster 2. In this subset, Cluster 2 had a hazard ratio for a subsequent asthma exacerbation requiring systemic corticosteroids of 2.71 [95% CI 1.11–6.64, *p* = 0.029] compared to Cluster 1 (Fig. 3). After adjusting for obesity, we found that Cluster 2 had an adjusted hazard ratio for a subsequent asthma exacerbation requiring systemic corticosteroids of 2.91 [95% CI 1.12–7.56, *p* = 0.028, *n* = 59] Although the proportion of children in Cluster 1 compared to Cluster 2 who had any medical visit for an asthma exacerbation in the year following discharge was higher for Cluster 2, this result did not reach statistical significance (24% vs. 70%, *p* = 0.063). There was no difference in the rates of hospitalization or inpatient admission, nor was there a difference in the number of asthma care visits or the number of Asthma/Allergy Clinic visits between the two clusters. There were six (24%) children in Cluster 1 and seven (30%) children in Cluster 2 with two or more exacerbations (*p* = 0.455) in the year following discharge from the hospital (Table 3).

**Table 3:** Duration of hospitalization and outcomes following hospitalization.

Characteristic, n (%)	Cluster 1 *n* = 41	Cluster 2 *n* = 28	*p-*value
Length of stay, median [IQR]			
ICU (days)	3.0 [2.0, 3.0]	3 [2.0, 3.0]	0.304
Hospital (days)	3.0 [3.0, 4.0]	3 [3.0, 4.0]	0.572
Discharged to			
Home	39 (100)	27 (100)	> 0.9
Inpatient rehabilitation	1 (2.4)	0 (0)	> 0.9
Discharge medications			
Albuterol or Xopenex	41 (100)	28 (100)	> 0.9
Montelukast	18 (44)	17 (61)	0.260
ICS	25 (61)	14 (50)	0.512
ICS + LABA	18 (44)	11 (39)	0.894
Oral corticosteroid burst	30 (73)	19 (68)	0.836
Dexamethasone	3 (10)	2 (11)	
Prednisolone	15 (50)	4 (21)	
Prednisone	12 (40)	13 (72)	
Daily oral corticosteroids	25 (61)	13 (46)	0.344
Primary outcome within 1 year	*n* = 33	*n* = 23	
Any medical visit for asthma exacerbation	8 (24)	12 (52)	0.063
Multiple asthma exacerbations	6 (18)	7 (30)	0.455
Secondary outcome within 1 year			
Number of exacerbations, median [IQR]	0.0 [0.0, 0.0]	0.0 [0.0, 2.0]	0.095
Follow-up location			
Urgent care/emergency department	8 (24)	12 (52)	0.063
Asthma/allergy clinic	14 (42)	16 (70)	0.083
# Asthma/allergy clinic visits, median [IQR]	0.0 [0.0, 2.0]	1.0 [0.0, 3.0]	0.133
Hospitalized, n (%)	6 (18)	7 (30)	0.455
# Inpatient admissions, median [IQR]			
General ward	0.0 [0.0, 0.0]	0.0 [0.0, 0.0]	> 0.9
ICU	0.0 [0.0, 0.0]	0.0 [0.0, 0.0]	0.727

### Cytokine and gene expression differences by cluster assignment

**Figure 4:**
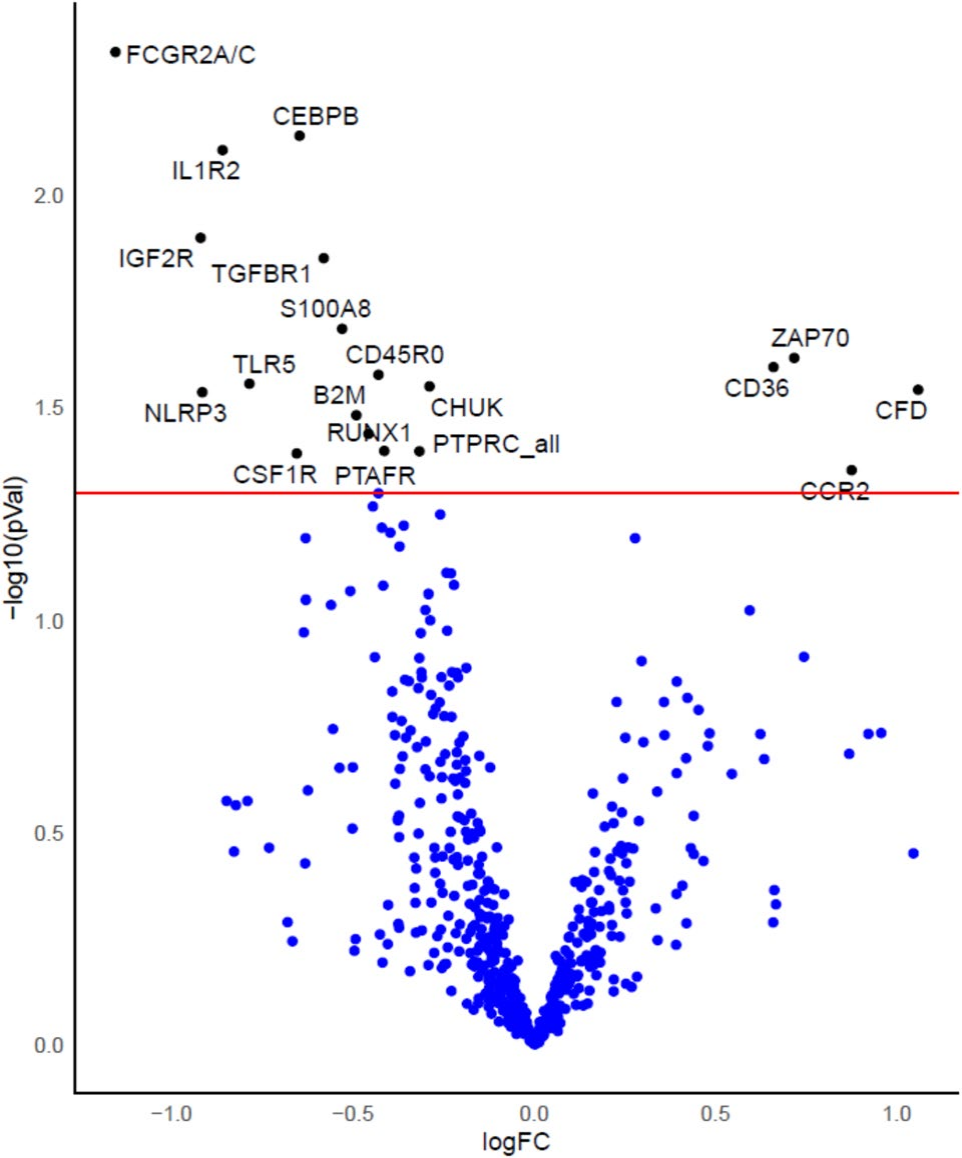
Volcano plot of the differentially expressed genes for Cluster 1 versus Cluster 2. There were 19 differentially expressed genes by cluster with 4 genes up-regulated and 15 genes down-regulated in Cluster 2 compared to Cluster 1. Each circle is a gene. Blue circles represent genes that not differentially expressed. Black circles represent genes with a false discovery rate of < 0.05. The horizontal red line is the y-axis threshold of log10[*p*-value] < 0.05.

Forty (58%) samples had neutrophil gene expression data available for analysis with 25 samples available from Cluster 1 and 15 available from Cluster 2 (Supplementary Fig. 1). We used a human immunology Nanostring array and analyzed differential gene expression data by assigned cluster. Because of the exploratory nature of our study, we did not correct for multiple comparisons. There were 19 differentially expressed genes by cluster with 4 genes up-regulated and 15 genes down-regulated in Cluster 2 compared to Cluster 1 (Fig. 4).

### Differential gene expression pathway analysis by cluster assignment

We next performed an exploratory analysis to visualize the neutrophil gene pathways that differed between the clusters using Reactome, excluding disease pathways^21,22^. The 26 pathways with at least one submitted entity found by gene over-representation analysis (false discovery rate (FDR) < 0.05) are sorted by ascending FDR value and shown in Supplementary Table 2. The top pathways included interleukin-10 signaling; nucleotide-binding domain, leucine rich repeat containing receptor (NLR signaling); and toll-like receptor (TLR) signaling.

## Discussion

In this study, we identified two subgroups or clusters of children with asthma admitted to a PICU who were distinguished by differences in their plasma cytokine concentrations, with Cluster 2 having lower cytokine levels than Cluster 1. Although there were essentially no clinical differences between these Clusters at PICU admission, children in Cluster 2 had 2.7-times the hazard of a recurrent asthma exacerbation in the year following PICU admission compared to children in Cluster 1. Exploratory analysis of purified blood neutrophils also identified differences in interleukin-10 signaling, NLR signaling, and TLR signaling between Clusters. Although these findings require additional validation, they highlight the variability in children admitted to a PICU for asthma and suggest that inflammatory mechanisms in this population warrant further study, particularly given the differing times to subsequent exacerbation between these two groups.

Asthma is a heterogeneous clinical syndrome with variability in symptom severity and disease trajectory^25^. To our knowledge, this is one of the first studies to characterize inflammatory differences in children admitted to a PICU for asthma and to relate those differences to longitudinal outcomes. Other studies have examined differences in children with acute asthma exacerbations as a whole compared children with stable asthma, but we do not know whether children with severe acute asthma exacerbations are comprised of multiple clinical phenotypes, nor do we know the relationship of a phenotype to underlying disease mechanisms and pharmacological treatment responses^11^. For example, one prior study of children presenting to an Emergency Department for an acute asthma exacerbation found differential expression of several immune-related genes including complement C3a receptor, integrin α4 (CD49d), arginase, Suppressor of Cytokine Signaling 3 (SOCS3), and interferon-mediated antiviral response genes in the transcriptomic profiles of nasal epithelial samples from the group as a whole^26^. However, we postulate that there may be significant variability within this population that has not been fully explored. Indeed, children with severe refractory asthma, who frequently have asthma exacerbations necessitating hospitalization, differ markedly in symptom burden, atopic features, comorbidities, airflow obstruction, and treatment responses^27^.

Because we generated clusters based on plasma cytokine levels, the two clusters that we identified were expected to have differing cytokine concentrations. However, inspection of the individual cytokines revealed that nearly all cytokines were higher in Cluster 1 versus Cluster 2, with the exception of IL-8, which did not differ and MIP-1A which had a lower median concentration. This was somewhat surprising since we anticipated that the clustering might identify children with a Type-2-high (T2-high) “atopic/allergic” subtype and a Type-2-low (T2-low) subtype, which are mutually exclusive^28^. Increased expression of IL-4, IL-5, and IL-13 are the hallmark cytokines of T2-high inflammation^29–31^, with decreased IL-2, IL-12, and IFNγ^28^. In contrast, T2-low asthma is poorly defined and relies on the absence of a T2-high signature^32^. While our clustering algorithm failed to identify T2-high and T2-low groups, other studies of severe refractory asthma have also suggested that there is no clear Th1 or Th2 profile in this group as a whole^33^. There is also evidence that Th1 cytokines increase in response to exacerbation triggers, which might account for increased levels of these cytokines in Cluster 1^34^.

We chose to focus our subtyping strategy on children admitted to the PICU for an asthma exacerbation rather than subtyping all children with asthma. As a result, we would expect that subtyping all children with asthma would not give the same clusters. We speculate that children with critical asthma may in fact be a further refinement of one of the existing asthma subtypes. To investigate this further, we would need to follow children with critical asthma as outpatients and enroll exacerbation prone children with asthma as a comparison group to understand the distinguishing immunologic, plasma cytokine, and transcriptomic features in children with versus without critical asthma. Furthermore, we believe that defining the immunologic features of children with critical asthma during an acute, life-threatening exacerbation compared to a quiescent state is an important step in understanding the immune and metabolic dysregulation of critical asthma.

Although not specifically assessed in the present study, viral infections are the primary trigger of acute asthma exacerbations necessitating hospitalization in children^35–39^. Guajardo and colleagues compared the differential gene expression profiles in the nasal respiratory epithelium of children having an acute asthma attack to that of children with non-exacerbating (stable) asthma and defined eight gene clusters that differentiated the clinical subgroups^26^. Children with exacerbating asthma showed the strongest and most reproducible gene expression signatures distinct from children with non-exacerbating asthma. Immune-related genes including complement 3a (C3a) receptor 1, IFN genes, integrin α4 (CD49d), chemokines such as CXCL11, and chemokine receptors were up-regulated in children with exacerbating asthma compared to children with non-exacerbating asthma^26^. Because all the children in the present study were admitted to the PICU, it is therefore not surprising that these genes were not significant. Instead, we analyzed gene expression from circulating neutrophils, similar to Su and colleagues^40^. In Cluster 1 with higher cytokine levels, we also found increased expression of NLRP3 (NLR and pyrin domain containing 3), which is associated with asthma severity^41,42^. NLRP3 activation also results in the activation and release of IL-1β, which was also higher in Cluster 1. Cluster 1 also showed increased expression of IL1R2 (interleukin 1 receptor type 2) a decoy receptor that inhibits IL-1β signaling. IL1R2 has increased expression in adults with allergic asthma compared to those with nonallergic asthma and is positively associated with severity^43^.

Our study has several limitations. First, this study was conducted in a PICU from a single center with a limited number of participants, limiting generalizability. Second, not all children had nasal swabbing performed to identify viral infections by PCR testing. Viral PCR testing was restricted for many months during the height of the COVID-19 pandemic due to limited supplies of nasal swabs and viral media needed to perform COVID-19 testing. Because not every child was tested for a viral respiratory infection, we cannot determine whether respiratory viruses were the inciting trigger for the exacerbation in enrolled participants. Third, we are limited to searching the electronic health records of the Children’s Healthcare of Atlanta system to determine whether children returned with an exacerbation within a year of hospital discharge; we cannot determine whether children received care outside of our healthcare system for an acute asthma attack. This approach also prohibits assessment of adherence to prescribed medications and access to asthma care, which could influence longitudinal asthma outcomes. Finally, many of the relevant gene pathways identified by Reactome were broadly related to the immune system or neutrophil biology. This is to be expected given that we ran a specific immune panel on isolated neutrophils.

## Conclusion

In summary, we have identified two clusters of children with asthma admitted to a PICU through plasma cytokine analysis that have differing times to exacerbation in the year following index hospitalization. The cluster with the higher hazard of exacerbation had lower concentrations across all cytokines tested. This observation warrants further study and suggests that a subset of children with recurrent asthma exacerbations may have a differing pattern of inflammation during the course of the hospitalization that may require alternative treatment approaches.

## Supplementary Information

Supplementary Information.

## Data Availability

The datasets generated and analyzed during the current study are available in the Gene Expression Omnibus (GEO) repository at under the accession number GSE208220 using the persistent weblink https://www.ncbi.nlm.nih.gov/geo/query/acc.cgi?acc=GSE208220.

## Abbreviations

ACT: Asthma Control Test
C-ACT: Childhood-Asthma Control Test
CBC: Complete blood count
EMR: Electronic Medical Record
EDTA: Ethylenediaminetetraacetic acid
GMCSF: Granulocyte-macrophage colony-stimulating factor
IRB: Institutional Review Board
CD49d: Integrin α4
IFN: Interferon
IFNG: Interferon gamma
ITAC: Interferon-inducible T cell alpha chemoattractant
IL: Interleukin
IL1R2: IL1 receptor type 2
MIP: Macrophage inflammatory protein
NLR: Nucleotide-binding domain, leucine rich repeat containing receptor
NLRP3: NLR and pyrin domain containing 3
PICU: Pediatric intensive care unit
PBS: Phosphate buffered saline
SARP: Severe Asthma Research Program
SOCS3: Suppressor of Cytokine Signaling 3
TLR: Toll-like receptor
TNFa: Tumor necrosis factor alpha
UMAP: Uniform manifold approximation and projection
U.S.: United States
