# Supplementary material for: Cluster analysis of plasma cytokines identifies two unique endotypes of children with asthma in the pediatric intensive care unit
Source: Sci Rep. 2023 Mar 2;13:3521. doi: 10.1038/s41598-023-30679-9 (PMC9978291; doi:10.1038/s41598-023-30679-9)
Supplement: Supplementary file 1 — Supplementary Information. [file 41598_2023_30679_MOESM1_ESM.docx]

**Cluster Analysis of Plasma Cytokines Identifies two Unique Endotypes of Children with Asthma in the Pediatric Intensive Care Unit**

Kirsten A. Cottrill, PhD, Milad G. Rad, MS, Michael J. Ripple, MD, PhD, Susan T. Stephenson, PhD, Ahmad F. Mohammad, BS, Mallory Tidwell, BSN, RN, Rishikesan Kamaleswaran, PhD, Anne M. Fitzpatrick, PhD, and Jocelyn R. Grunwell, MD, PhD

**Online Data Supplement**

**Supplementary Figure 1.** Flow Diagram of Participants Enrolled and Analyzed in the Study.

**Supplementary Table 1.** Emergency Department and ICU Treatments, Laboratory Study Results

**Supplementary Table 2.** Reactome pathways with >1 submitted entity found sorted by FDR

**Supplementary Figure 1.** Flow Diagram of Participants Enrolled and Analyzed in the Study.

**
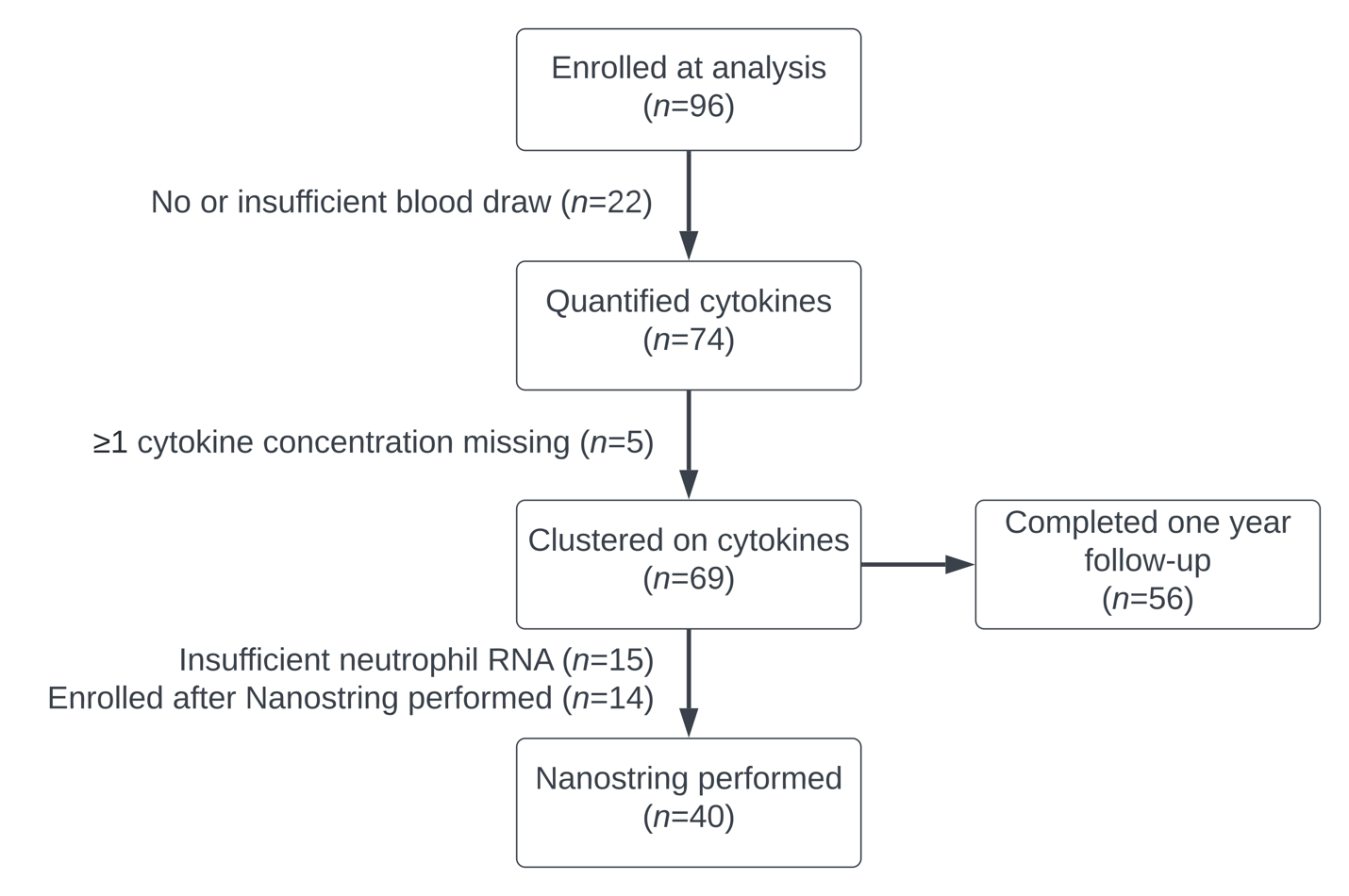
**

**Supplementary Table 1. Emergency Department and ICU Treatments, Laboratory Study Results**

| Characteristic, n (%) | Cluster 1  *n* = 41 | Cluster 2  *n* = 28 |  | *p-*value |
| --- | --- | --- | --- | --- |
| Medications Given Prior to PICU  Intermittent albuterol  Number, mean (SD)  Continuous albuterol  Magnesium sulfate (IV)  Corticosteroids  Inhaled  Oral  IV  Epinephrine (IM/SQ)  Terbutaline | 4 (9.8)  2.8 (2.4)  41 (100)  38 (93)  18 (44)  15 (37)  16 (39)  7 (17)  5 (12) | 6 (21)  5.0 (5.7)  26 (93)  24 (86)  9 (32)  5 (18)  14 (50)  4 (14)  1 (3.6) |  | 0.296  0.453  0.161  0.430  0.464  0.157  0.512  >0.9  0.389 |
| Medications Given in PICU  Continuous albuterol  Magnesium sulfate (IV)  Terbutaline  Heliox (80/20)  Aminophylline  Isoflurane | 36 (88)  19 (50)  11 (27)  7 (18)  2 (4.9)  3 (7.5) | 23 (82)  8 (32)  3 (11)  6 (21)  1 (3.7)  0 (0) |  | 0.729  0.249  0.207  0.869  >0.9  0.267 |
| Ventilatory Support in ICU  No respiratory support  High-flow nasal cannula  Non-invasive positive pressure ventilation  Invasive mechanical ventilation  Isoflurane ventilator | 5 (12)  14 (34)  25 (61)  4 (9.8)  3 (7.3) | 2 (7.1)  10 (36)  14 (50)  2 (7.1)  0 (0) |  | 0.693  >0.9  0.512  >0.9  0.266 |
| CBC with Differential Performed  WBC count (10^9^/L), mean (SD)  Absolute neutrophil count, mean (SD)  Absolute eosinophil count, mean (SD) | 10 (24)  17.1 (6.8)  14.0 (6.9)  0.4 (0.3) | 8 (29)  15.3 (3.7)  13.0 (4.1)  0.3 (0.3) |  | >0.9  0.484  0.703  0.584 |

**Supplementary Table 2. Reactome pathways with >1 submitted entity found sorted by FDR**

|  | Entities | | | Reactions | |
| --- | --- | --- | --- | --- | --- |
| Pathway name | **Found** | **Ratio** | **FDR^a^** | **Found** | **Ratio** |
| Immune System | 22 | 1.78E-01 | 2.20E-07 | 160 | 1.18E-01 |
| Interleukin-10 signaling | 6 | 5.69E-03 | 4.50E-06 | 2 | 1.09E-03 |
| Signaling by Interleukins | 10 | 4.27E-02 | 6.80E-05 | 14 | 3.57E-02 |
| Diseases associated with the TLR signaling cascade | 4 | 2.78E-03 | 8.70E-05 | 10 | 1.09E-03 |
| Diseases of Immune System | 4 | 2.78E-03 | 8.70E-05 | 10 | 1.09E-03 |
| Cytokine Signaling in Immune system | 12 | 7.24E-02 | 8.70E-05 | 21 | 5.15E-02 |
| Innate Immune System | 13 | 8.86E-02 | 8.70E-05 | 102 | 5.15E-02 |
| Neutrophil degranulation | 8 | 3.18E-02 | 2.40E-04 | 7 | 7.25E-04 |
| Transcriptional regulation of granulopoiesis | 4 | 4.70E-03 | 5.40E-04 | 9 | 1.96E-03 |
| Transcriptional regulation of white adipocyte differentiation | 4 | 7.22E-03 | 2.30E-03 | 6 | 1.30E-03 |
| Transcriptional Regulation by VENTX | 3 | 3.18E-03 | 3.60E-03 | 4 | 9.42E-04 |
| Nucleotide-binding domain, leucine rich repeat containing receptor (NLR) signaling pathways | 3 | 4.77E-03 | 1.00E-02 | 9 | 3.33E-03 |
| ER-Phagosome pathway | 4 | 1.15E-02 | 1.00E-02 | 4 | 7.25E-04 |
| Toll-like Receptor Cascades | 4 | 1.25E-02 | 1.40E-02 | 41 | 1.34E-02 |
| Antigen processing-Cross presentation | 4 | 1.29E-02 | 1.40E-02 | 9 | 1.67E-03 |
| MyD88 deficiency (TLR2/4) | 2 | 1.72E-03 | 2.10E-02 | 2 | 1.45E-04 |
| IRAK4 deficiency (TLR2/4) | 2 | 1.79E-03 | 2.10E-02 | 2 | 1.45E-04 |
| Toll Like Receptor TLR6:TLR2 Cascade | 3 | 7.88E-03 | 2.20E-02 | 24 | 4.78E-03 |
| MyD88:MAL(TIRAP) cascade initiated on plasma membrane | 3 | 7.88E-03 | 2.20E-02 | 22 | 4.64E-03 |
| Toll Like Receptor 2 (TLR2) Cascade | 3 | 8.08E-03 | 2.20E-02 | 24 | 4.93E-03 |
| Toll Like Receptor TLR1:TLR2 Cascade | 3 | 8.08E-03 | 2.20E-02 | 22 | 4.78E-03 |
| Interferon gamma signaling | 4 | 1.66E-02 | 2.20E-02 | 2 | 1.16E-03 |
| Regulation of TLR by endogenous ligand | 2 | 2.38E-03 | 2.30E-02 | 2 | 8.70E-04 |
| TCR signaling | 3 | 9.73E-03 | 3.20E-02 | 12 | 3.77E-03 |
| Toll Like Receptor 4 (TLR4) Cascade | 3 | 1.01E-02 | 3.60E-02 | 23 | 6.89E-03 |
| Adaptive Immune System | 7 | 6.70E-02 | 3.90E-02 | 49 | 1.91E-02 |

^a^False Discovery Rate
